# Structures and Energetics of E_2_H_3_^+^ (E = As, Sb, and Bi) Cations

**DOI:** 10.1021/acs.jpca.3c05945

**Published:** 2024-01-16

**Authors:** Shu-Hua Xia, Jihuan He, Zhuoqun Liu, Yunhan Liu, Yan Zhang, Yaoming Xie, Mitchell E. Lahm, Gregory H. Robinson, Henry F. Schaefer

**Affiliations:** † College of Life and Environmental Sciences, Minzu University of China, Beijing 100081, China; ‡ Department of Chemistry and Center for Computational Quantum Chemistry, University of Georgia, Athens, Georgia 30602, United States

## Abstract

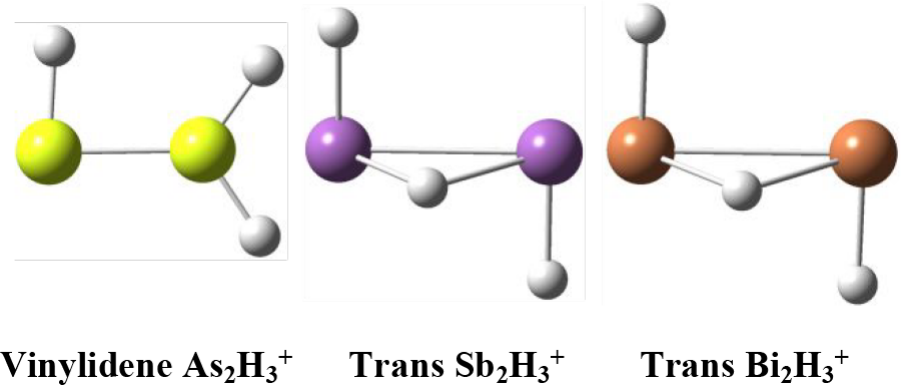

E_2_H_2_ (E = As, Sb, Bi) structures involving multiple bonds have attracted much attention recently. The E_2_H_3_^+^ cations (protonated E_2_H_2_) are predicted to be viable with substantial proton affinities (>180 kcal/mol). Herein, the bonding characters and energetics of a number of E_2_H_3_^+^ isomers are explored through CCSD(T) and DFT methods. For the As_2_H_3_^+^ system, the CCSD(T)/cc-pVQZ-PP method predicts that the vinylidene-like structure lies lowest in energy, with the trans and cis isomers higher by 6.7 and 9.3 kcal/mol, respectively. However, for Sb_2_H_3_^+^ and Bi_2_H_3_^+^ systems, the trans isomer is the global minimum, while the energies of the cis and vinylidene-like structures are higher, respectively, by 2.0 and 2.4 kcal/mol for Sb_2_H_3_^+^ and 1.6 and 15.0 kcal/mol for Bi_2_H_3_^+^. Thus, the vinyledene-like structure is the lowest energy for the arsenic system but only a transition state of the bismuth system. With permanent dipole moments, all minima may be observable in microwave experiments. Besides, we have also obtained transition states and planar-cis structures with higher energies. The current results should provide new insights into the various isomers and provide a number of predictions for future experiments.

## Introduction

Main-group chemistry involving multiple bonds between heavier elements is a rapidly developing field.^1−7^ Particularly, the compounds of group 15 elements, such as the dipnictogen HEEH (E = N, P, As, Sb, Bi) molecules, have been studied systematically.^8,9^ Among these, in comparison with the diazenes and diphosphenes, the heavier congeners diarsenes, distibenes, and dibismuthenes are less frequently reported, showing the difficulties in stabilizing multiple bonds between the heavier atoms. In 1997, Tokitoh, Arai, Okazaki, and Nagase synthesized the first stable dibismuthene, in which a Bi=Bi double bond was characterized by means of UV–vis and Raman spectra, X-ray crystallographic structural analysis, and computations.^10^ In 1998, the same research group synthesized the stable distibene (RSb=SbR).^11,12^ Later, Schulz et al. determined the solid-state structures of Et_4_Sb_2_ and Et_4_Bi_2_ using X-ray diffraction.^13^ In 2015, Scheer and co-workers reported the isolation and structural authentication of HAsAsH in a bulky diuranium(IV) complex.^14^ The same group in 2019 incorporated the HAs=AsH moiety as a side-on-coordinated ligand in a simple mononuclear Fe(CO)_4_ complex.^15^

Theoretically, as early as 1990, Nagase, Suzuki, and Kurakake predicted that the Sb and Bi atoms can form double-bonded compounds at the HF/DZ(d,p) level of theory.^16^ In 2008 and 2014, Su et al. explored the lowest singlet and triplet potential energy surfaces for the HAsXH and HSbXH (X = N, P, As, Sb, Bi) systems with the QCISD/LANL2DZdp method, and their results are in good agreement with the limited experimental results.^17,18^ In 2020, Li, Huang, Xie, Robinson, and Schaefer investigated the E_2_H_2_ (E = As, Sb, Bi) molecules with the CCSD(T) method as well as four different density functional theory (DFT) methods.^19^ They found that the trans isomer lies lowest in energy, but both trans and cis structures may be observable due to large barriers between them. Their natural bond orbital (NBO) analyses showed that, as expected, the Wiberg bond indices (WBIs) for the E=E bond in the trans, cis, and vinylidene-like structures are all close to 2.0.

Related viable cations are appealing synthetic targets. It is common knowledge that for alkenes the C=C double bond can be protonated to form carbocations. In 2016, the protonation of disilicon(0) compounds was reported by Filippou et al.,^20^ and a topomerization was found between the “σ-bonded” protonation tautomer and the “π-bonded” disilahydronium ion. For the group 15 elements, the methylation of a diphosphene (Mes*P=PMes*) was reported to form a stable phosphanyl phosphenium cation.^21^ However, the corresponding protonation of the diphosphene was not observed.^22^ To our knowledge, the cations designed by adding a proton to the E_2_H_2_ (E = As, Sb, Bi) molecules have not been reported. For these reasons, these cations are worthy of study. Hence, we examine the structural features of protonated E_2_H_3_^+^ (E = As, Sb, Bi) in the present research.

## Methodology

Optimized geometries and energies of all E_2_H_3_^+^ (E = As, Sb, Bi) minima and transition states (TSs) were initially obtained employing four DFT methods (BP86, B3LYP, ωB97X-D, and MN15).^23−26^ This preliminary research was followed by optimizations with the “gold standard” coupled cluster single and double with perturbative triple excitations [CCSD(T)] method.^27−29^ For the H atoms, Dunning’s correlation-consistent polarized valence basis sets were used [cc-pVTZ with DFT and cc-pVQZ with the high-level CCSD(T)].^30,31^ For the As, Sb, and Bi atoms, we adopted the multiconfiguration Dirac–Hartree–Fock adjusted small-core relativistic pseudopotentials (PPs) in conjunction with the corresponding correlation-consistent basis sets of Peterson, i.e., cc-pVnZ-PP (n = T, Q).^32,33^ The vibrational frequency analyses were carried out at the same level of theory to verify all the obtained structures to be genuine minima or transition states. Intrinsic reaction coordinate (IRC)^34^ analyses were performed with the DFT methods to verify the minima connecting the transition states.

While the results from all five methods are listed in the figures and tables, only the CCSD(T) results are discussed in the text. The DFT results can be used to assess the quality of a functional in comparison with the CCSD(T) method. The CCSD(T) computations were performed with MOLPRO 2010.^35^ The DFT computations were carried out with Gaussian 16.^36^

## Results

### As_2_H_3_^+^ Structures

**Figure 1:**
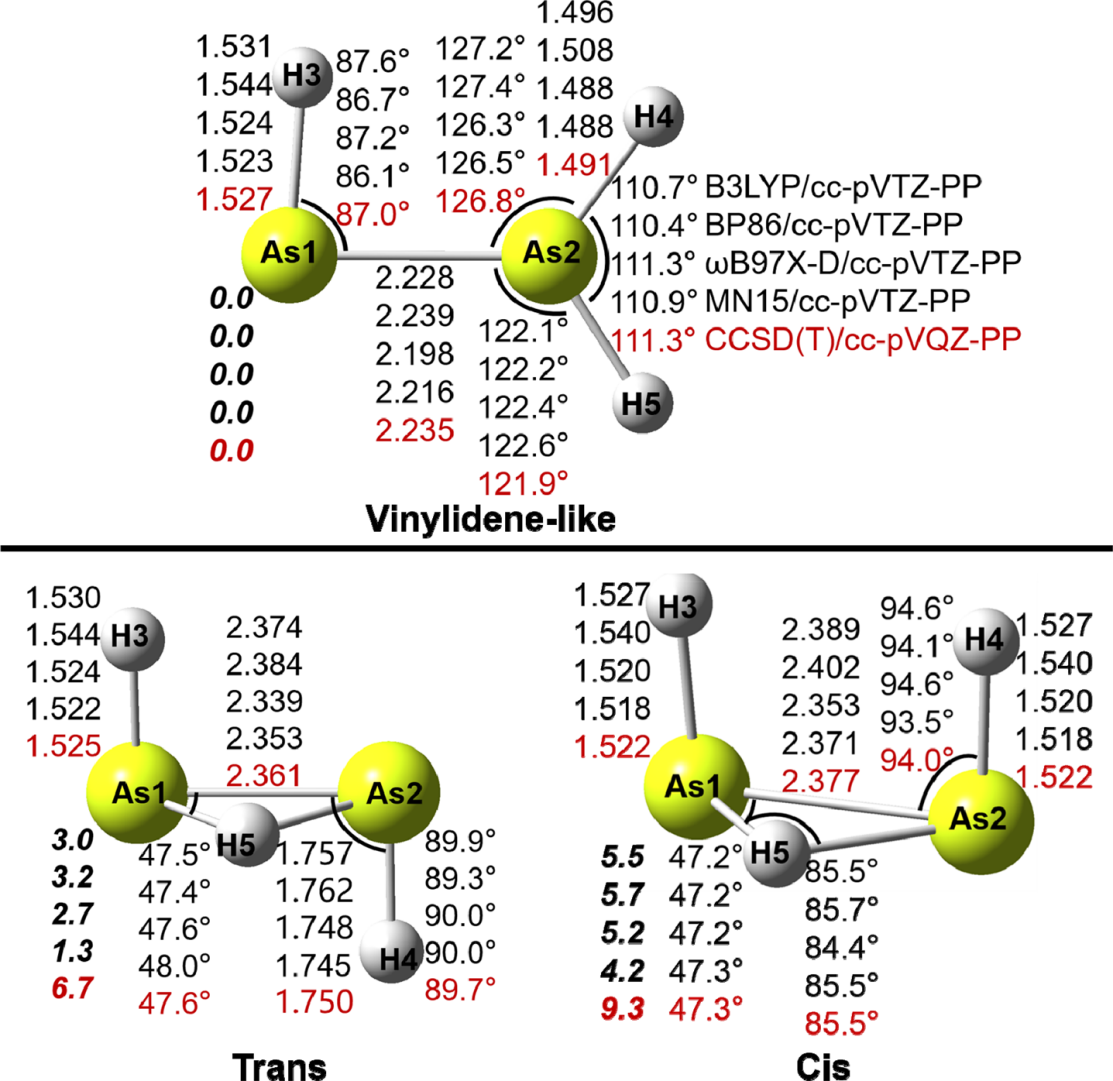
Geometries and energetics for three As_2_H_3_^+^ equilibrium geometries. Bond distances are in Å and relative energies (**bold face**) in kcal/mol.

We have optimized seven structures for the As_2_H_3_^+^ system. Among these are three genuine minima (trans, cis, and protonated vinylidene-like), three transition states (TS-T-V, TS-C-V, and TS-C-C), and a second-order saddle point (planar-cis). Figure 1 and Table 1 report geometries and relative energies of these structures.

**Table 1:** Relative Energies (Δ*E*, in kcal/mol) and As–As Bond Distances (*R*, in Å) for As_2_H_3_^+^ at the CCSD(T)/cc-pVQZ-PP Level of Theory and As–As Wiberg Bond Indices (WBIs) and As Atomic Charges (*Q*_As_) from Natural Bond Orbital (NBO) Analysis at the MN15/cc-pVTZ-PP Level

structure	Δ*E*	*R*_As–As_	WBI_As–As_	*Q*_As_
vinylidene	0.0	2.235	1.90	0.52/0.33
trans	6.7	2.361	1.28	0.57/0.57
cis	9.3	2.377	1.25	0.55/0.55
TS-T-V	10.8	2.363	1.37	0.76/0.27
TS-C-V	13.2	2.375	1.33	0.74/0.29
TS-C-C	29.3	2.397	1.52	0.47/0.47
planar-cis	60.3	2.209	2.08	0.35/0.35

**Figure 2:**
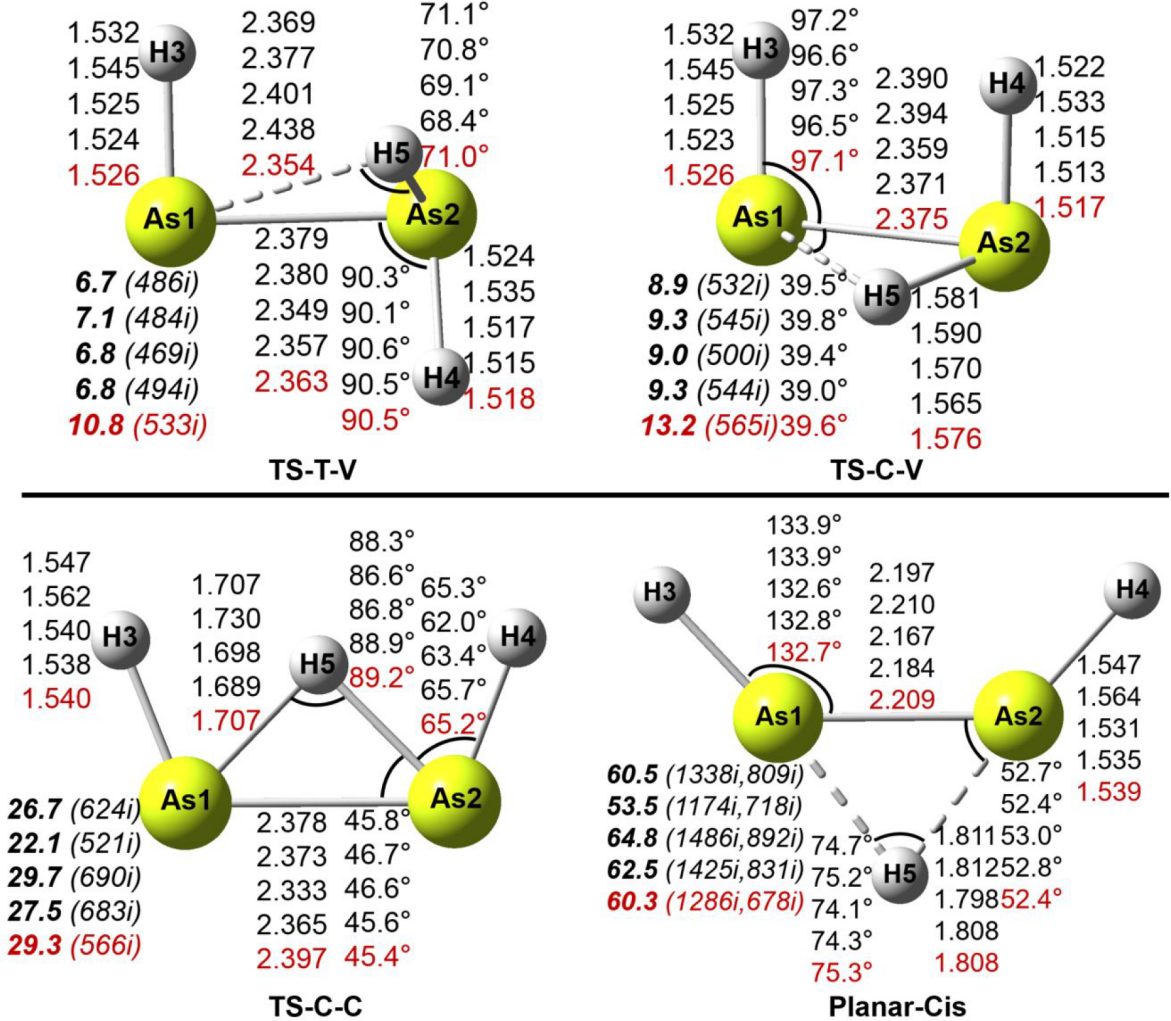
Geometries and energetics for four As_2_H_3_^+^ transition states. Bond distances are in Å and relative energies (**bold face**) to the vinylidene-like global minimum (in Figure 1) in kcal/mol. The imaginary vibrational frequencies (in cm^–1^) are shown in parentheses.

The relative energies predicted by the different DFT methods are in reasonable agreement with each other, but they are lower than those for the CCSD(T) results, except for planar-cis (Figures 1 and 2). The vinylidene structure is predicted to be lowest-lying, different from the energy order for neutral As_2_H_2_, for which the global minimum is the trans structure.^15,19^ The trans As_2_H_3_^+^ structure is predicted to lie 6.7 kcal/mol higher than the vinylidene structure by the CCSD(T)/cc-pVQZ-PP method, and the cis structure lies 9.3 kcal/mol above the vinylidene structure. Interestingly, the energy order of the As_2_H_3_^+^ isomers (vinylidene and trans) is similar to that for the observed protonated cation [Si_2_(H)(Idipp)_2_]^+^ of the disilicon(0) compound.^20^ The latter most favored structure is the Si–H “σ-bonded” minimum, while the “π-bonded” minimum is higher in energy by 7.3 kcal/mol. A transition state between them has a relative energy of 10.3 kcal/mol.^20^

For the TS-T-V transition state, the IRC analysis shows that it connects the trans and vinylidene structures with an energy barrier of 10.8 kcal/mol with respect to the vinylidene side and 4.1 kcal/mol from the trans side at the CCSD(T)/cc-pVQZ-PP level. The TS-C-V structure is a transition state connecting the cis and vinylidene structures with a barrier height of 3.9 kcal/mol from the cis minimum and 13.2 kcal/mol from the vinylidene-like side. The TS-C-C transition state is found to connect the two mirror images of the cis isomer with an energy barrier of 20.0 kcal/mol. The planar-cis structure has two imaginary vibrational frequencies with a very high relative energy (60.3 kcal/mol) above the vinylidene minimum.

Figure 1 shows that the DFT-optimized geometries for all As_2_H_3_^+^ structures are in qualitative agreement with the CCSD(T) results. The global-minimum vinylidene-like structure is planar, and its As–As bond distance is 2.235 Å (with the CCSD(T)/cc-pVQZ-PP method), which is 0.08 Å longer than that (2.154 Å) in the neutral vinylidene-like As_2_H_2_.^19^ The As–As bond distances in the trans and cis structures are predicted to be 2.361 and 2.377 Å, longer than that of the vinylidene-like structure by 0.13 and 0.14 Å, respectively. The CCSD(T)-predicted harmonic vibrational frequencies for As–As stretching are 259 (vinylidene), 321 (trans), and 312 (cis) cm^–1^, respectively (Table S3). The As–As distances for the transition states TS-C-C, TS-C-V, and TS-C-C are 2.363, 2.375, and 2.397 Å, respectively. The As–As bond distance in the planar-cis structure is the shortest, i.e., 2.209 Å, suggesting the strongest As=As double bond.

According to the NBO analyses, the As atoms in all As_2_H_3_^+^ isomers display positive charges, and the sum of charges on the two As atoms is close to +1 (Table 1), indicating that the added charge resides primarily in the vicinity of the two As atoms. The trans and cis structures are “π-bonded” isomers, which makes the proton attract electron density from the As–As π-bonding orbital, leading to a lower As–As bond order. Table 1 shows that the WBI values for the As–As bonds for the As_2_H_3_^+^ trans and cis isomers are 1.28 and 1.25, respectively, much lower than their corresponding WBI values for the As_2_H_2_ trans (2.03) and cis (2.01) isomers.^19^ The vinylidene structure is a “σ-bonded” isomer, which makes the proton attract electron density from an As lone pair orbital, with little effect on the As=As double bond. Thus, the corresponding WBI value is 1.90 (Table 1), comparable with the WBI value (1.99) for the neutral As_2_H_2_ vinylidene isomers.^19^ Obviously, these As–As WBI values are consistent with the As–As bond distances (Table 1). Accordingly the As–As WBI values for the transition states are 1.37 (TS-T-V), 1.33 (TS-C-V), and 1.52 (TS-C-C), while that for planar-cis is 2.08 (Table 1).

### Sb_2_H_3_^+^ Structures

**Figure 3:**
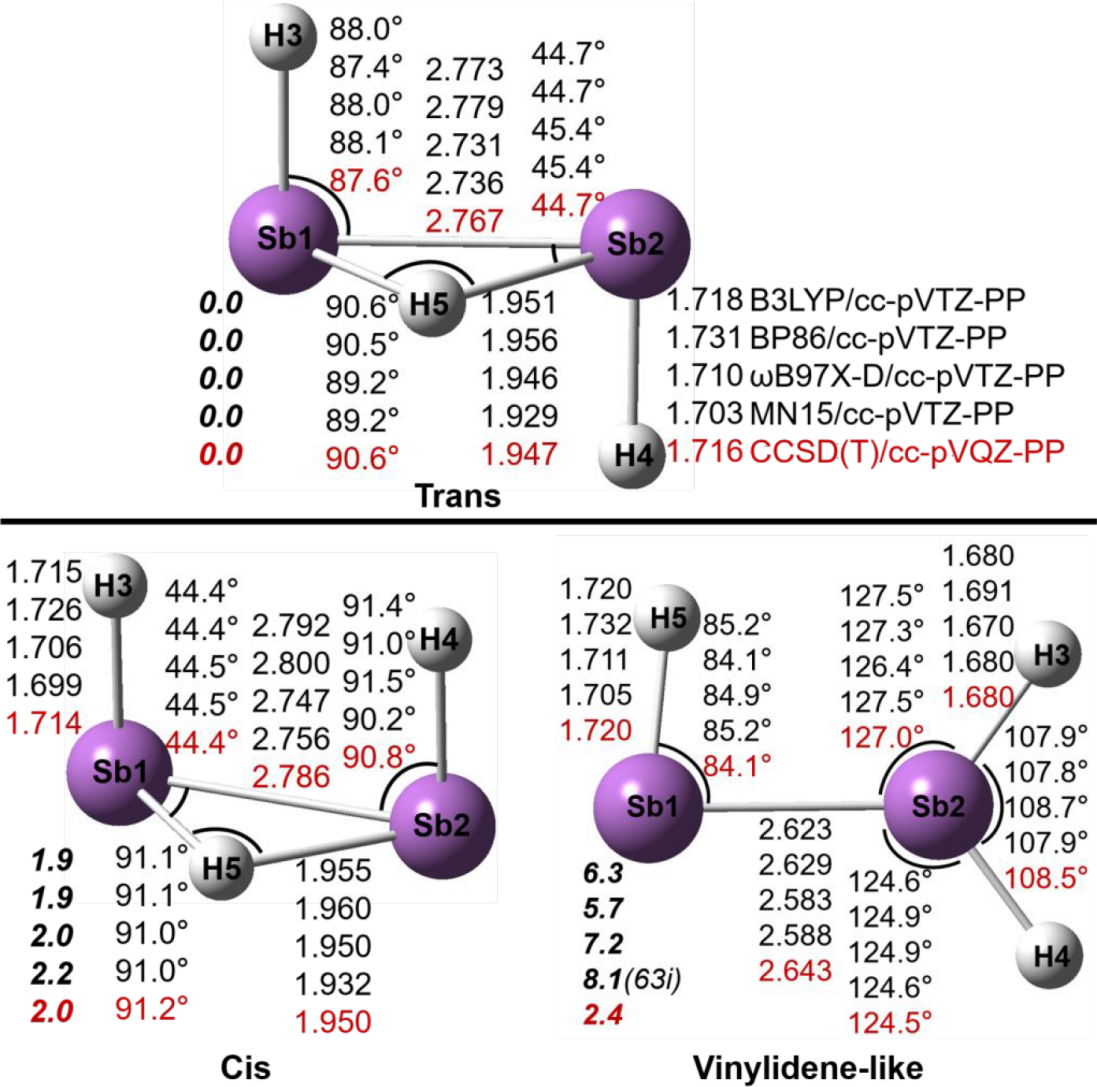
Geometries and energetics for three Sb_2_H_3_^+^ equilibrium geometries. Bond distances are in Å and energies (**bold face**) in kcal/mol.

**Figure 4:**
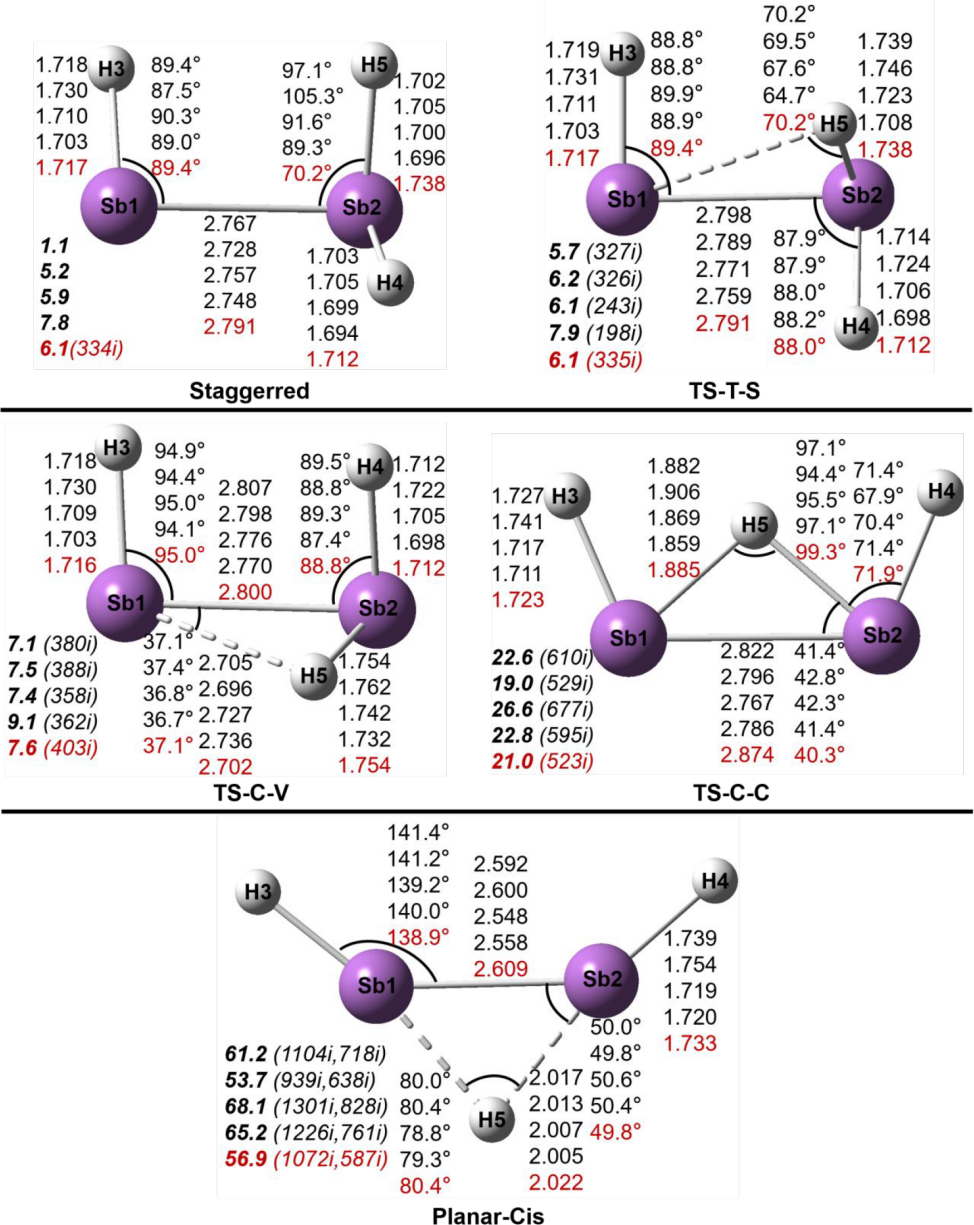
Geometries and energetics for five Sb_2_H_3_^+^ transition states. Bond distances are in Å and energies (**bold face**) relative to the trans global minimum (in Figure 3) in kcal/mol. The imaginary vibrational frequencies (in cm^–1^) are shown in parentheses.

We have found eight structures for the Sb_2_H_3_^+^ system (Figures 3 and 4 and Table 2). Like As_2_H_3_^+^ in the previous section, the trans, cis and vinylidene-like structures are predicted to be genuine minima. However, unlike As_2_H_3_^+^ the lowest-lying Sb_2_H_3_^+^ structure is the trans structure with the added proton “π-coordinated”. The cis and vinylidene Sb_2_H_3_^+^ structures lie above the trans structure by 2.0 and 2.4 kcal/mol, respectively. For Sb_2_H_3_^+^, a staggered minimum is also found, but only by the DFT methods, lying 7.8 kcal/mol above the trans structure at the MN15/cc-pVTZ-PP level. However, with the CCSD(T)/cc-pVQZ-PP method, the staggered structure is a transition state connecting the trans and vinylidene minima. The DFT methods located a transition state TS-T-S connecting the trans and staggered structures with only a tiny energy barrier from the staggered side (<0.2 kcal/mol with the ωB97X-D and MN15 methods). Another transition state, TS-C-V, is found between the cis and vinylidene minima, lying 5.2 kcal/mol in energy above vinylidene and 5.6 kcal/mol above cis. The TS-C-C structure is a transition state connecting two mirror cis minima with a high barrier (19.0 kcal/mol). The planar-cis structure is a second-order stationary point (with two imaginary vibrational frequencies, 1072i and 586i cm^–1^), and it lies 56.9 kcal/mol above the trans structure, comparable to that (60.3 kcal/mol) for the planar-cis structure of the analogous As_2_H_3_^+^ system.

**Table 2:** Relative Energies (Δ*E*, in kcal/mol) and Sb–Sb Bond Distances (*R*, in Å) for Sb_2_H_3_^+^ at the CCSD(T)/cc-pVQZ-PP level of theory and Sb–Sb Wiberg Bond Indices (WBIs) and Sb Atomic Charges (*Q*_Sb_) from Natural Bond Orbital (NBO) Analysis at the MN15/cc-pVTZ-PP Level

structure	Δ*E*	*R*_Sb–Sb_	WBI_Sb–Sb_	*Q*_Sb_
trans	0.0	2.767	1.21	0.70/0.70
cis	2.0	2.786	1.19	0.69/0.69
vinylidene	2.4	2.643	1.84	0.63/0.46
staggered^a^	7.8	2.748	1.34	0.85/0.37
TS-T-S	6.1	2.791	1.29	0.87/0.39
TS-C-V	7.6	2.800	1.24	0.87/0.43
TS-C-C	21.0	2.874	1.36	0.63/0.63
planar-cis	56.9	2.609	2.01	0.52/0.52

a The values for the staggered structure are from the MN15 method, since it is not a minimum with the CCSD(T) method.

The Sb–Sb bond distances in the trans and cis Sb_2_H_3_^+^ structures are predicted to be 2.767 and 2.786 Å, respectively, longer than those in the corresponding neutral Sb_2_H_2_ structures by 0.14 Å.^19^ Accordingly, the Sb–Sb WBI values decrease from ∼2.0 for the neutral Sb_2_H_2_ molecules to ∼1.2 for the Sb_2_H_3_^+^ cations (Table 2). The decrease of the Sb–Sb bond order for the trans and cis structures arises because the added proton breaks the Sb–Sb π bond to form an Sb–H–Sb two-electron three-center (2e–3c) bond. The Sb–Sb bond distance in the vinylidene-like structure (2.643 Å) is shorter, and its WBI value is larger (1.84) (Table 2), since the added proton approaches a lone pair orbital, leaving the Sb–Sb π bond little affected. The planar-cis structure has the shortest Sb–Sb bond distance (2.609 Å), consistent with its largest WBI value (2.01) and its largest Sb–Sb stretching vibrational frequency (227 cm^–1^; Table S3), similar to the planar-cis structure of As_2_H_3_^+^. The transition states were found to have slightly longer Sb–Sb distances than their related minima, i.e., TS-T-S (2.791 Å), TS-C-V (2.800 Å), and TS-C-C (2.874 Å).

### Bi_2_H_3_^+^ Structures

**Figure 5:**
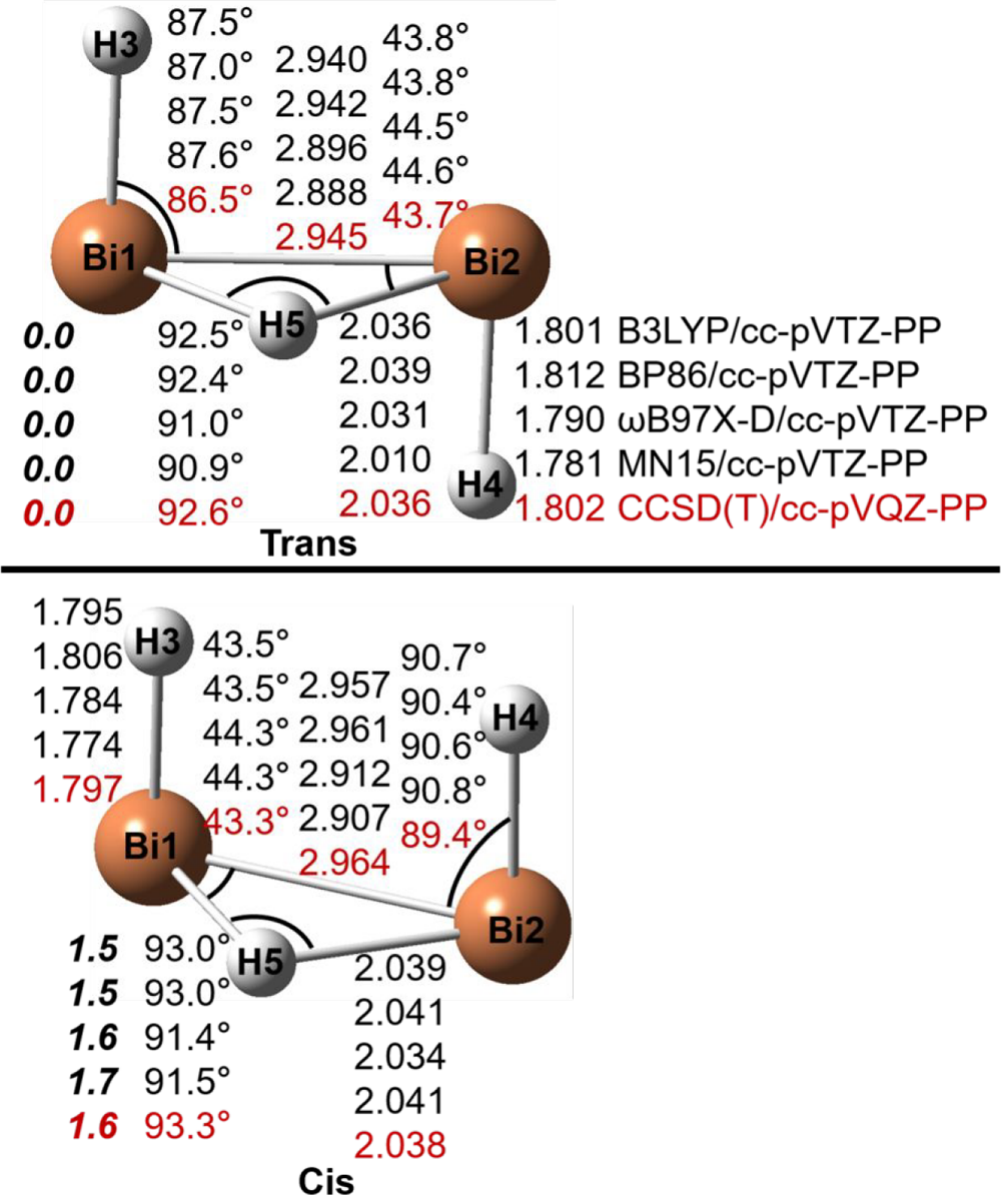
Geometries and energetics for two Bi_2_H_3_^+^ equilibrium geometries. Bond distances are in Å and energies (**bold face**) in kcal/mol.

**Figure 6:**
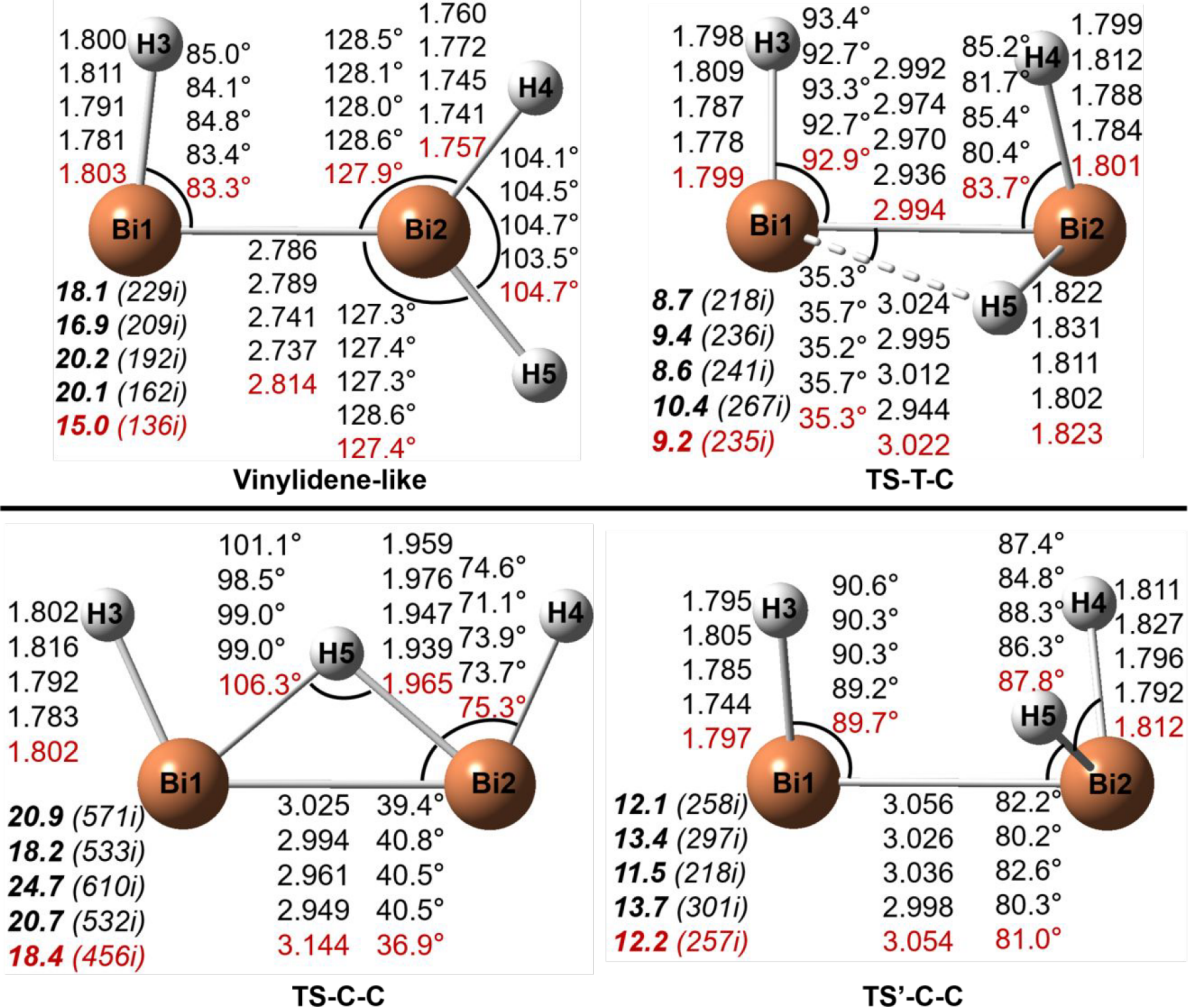
Geometries and energetics for four Bi_2_H_3_^+^ transition states. Bond distances are in Å and energies (**bold face**) relative to the trans global minimum (in Figure 5) in kcal/mol. The imaginary vibrational frequencies (in cm^–1^) are shown in parentheses.

The geometries of the Bi_2_H_3_^+^ stationary points are reported in Figures 5 and 6. Similar to Sb_2_H_3_^+^, the trans structure is the global minimum, while the cis structure is 1.6 kcal/mol above trans. Unlike the As and Sb analogues, the vinylidene-like structure for Bi_2_H_3_^+^ is a transition state predicted by all theoretical methods and lies 15.0 kcal/mol above trans with the CCSD(T) method. The normal mode corresponding to the imaginary vibrational frequency is the −BiH_2_ wag. The IRC analysis shows that the vinylidene transition state connects two mirror trans structures. Structure TS-T-C is a transition state between the trans and cis structures, lying 9.2 kcal/mol above trans. The TS-C-C structure, similar to those for Sb_2_H_3_^+^ and As_2_H_3_^+^, is a transition state connecting two mirror cis structures with the energy barrier of 16.8 kcal/mol, comparable to 19.0 kcal/mol for Sb_2_H_3_^+^ and 20.0 kcal/mol for As_2_H_3_^+^. Interestingly, we found another transition state, TS′-C-C, that also connects two mirror cis isomers with a lower energy barrier (10.6 kcal/mol). Unlike the analogous As_2_H_3_^+^ and Sb_2_H_3_^+^ systems, there is no stationary planar-cis saddle point for the Bi system, since it collapses to TS-C-C.

The Bi–Bi bond distance in the trans Bi_2_H_3_^+^ isomer is predicted to be 2.945 Å (Figure 3 and Table 3), much longer than that for neutral Bi_2_H_2_, 2.780 Å (theoretical)^19^ or 2.821 and 2.854 Å (experimental values from crystal structures).^13,31^ Similar to the case of trans As_2_H_3_^+^ and Sb_2_H_3_^+^, the added proton weakens the Bi–Bi π bond and decreases the Bi–Bi WBI value from 2.02 (neutral Bi_2_H_2_) to 1.20 (cationic Bi_2_H_3_^+^). Similarly, the Bi–Bi distance in the cis Bi_2_H_3_^+^ isomer is predicted to be 2.964 Å, longer than that for the neutral Bi_2_H_2_ (2.794 Å).^19^ The Bi–Bi WBI value is 1.18, smaller than that (2.01) for neutral Bi_2_H_2_.^19^ The vinylidene structure has a shorter Bi–Bi bond distance (2.814 Å) and larger WBI value (1.71), while the three transition states have longer Bi–Bi distances and smaller WBIs (Table 3).

**Table 3:** Relative Energies (Δ*E*, in kcal/mol) and Bi–Bi Bond Distances (*R*, in Å) for Bi_2_H_3_^+^ at the CCSD(T)/cc-pVQZ-PP Level of Theory and Bi–Bi Wiberg Bond Indices (WBIs) and Bi Atomic Charges (*Q*_Bi_) from Natural Bond Orbital (NBO) Analysis at the MN15/cc-pVTZ-PP Level

structure	Δ*E*	*R*_Bi–Bi_	WBI_Bi–Bi_	*Q*_Bi_
trans	0.0	2.945	1.20	0.72/0.72
cis	1.6	2.964	1.18	0.71/0.71
vinylidene	15.0	2.814	1.71	0.67/0.41
TS-T-C	9.2	2.994	1.16	0.89/0.45
TS-C-C	18.4	3.144	1.31	0.64/0.64
TS′-C-C	12.2	3.054	1.04	0.93/0.41

## Discussion

### Geometries and Energetics

**Figure 7:**
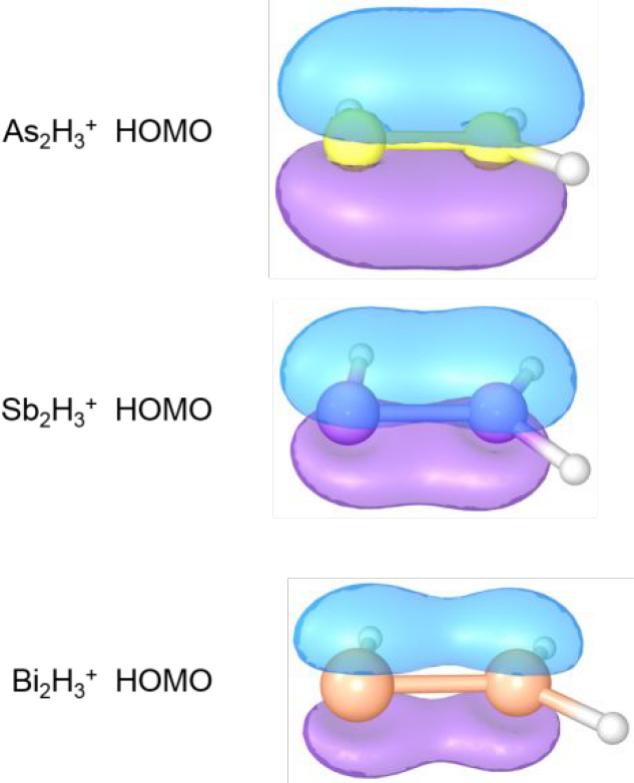
HOMO orbitals of the vinylidene-like structures of the As_2_H_3_^+^, Sb_2_H_3_^+^, and Bi_2_H_3_^+^ systems.

Compared with previous theoretical investigations on the neutral E_2_H_2_ molecules of group 15 (E = As, Sb, Bi),^19^ we find some similarities and some differences for the E_2_H_3_^+^ cations upon the addition of H^+^ to the neutral E_2_H_2_ molecules. For example, we find the trans and cis structures to be genuine minima for all three E_2_H_3_^+^ systems, with the cis structures lying always higher than the trans structures by ∼2 kcal/mol. This is similar to the situation for the neutral E_2_H_2_ molecules.^19^ However, with an added “σ-coordinated” E–H bond, the vinylidene-like structure is a global minimum for As_2_H_3_^+^, lying *below* the trans structure by ∼7 kcal/mol, while the vinylidene-like structure for Sb_2_H_3_^+^ is still a minimum but lies *above* the trans structure with a “π-coordinated” E–H bond by ∼2 kcal/mol. Finally, the vinylidene-like structure for Bi_2_H_3_^+^ is a transition state lying *above* the trans structure by ∼15 kcal/mol (Table 4). This should be attributed to the weaker Sb–Sb and Bi–Bi π bonds compared to the As–As π bond, following the periodic trend. A diagram of MOs (Figure 7) for the E–E π bonds (E = As, Sb, Bi) shows such a trend.

**Table 4:**
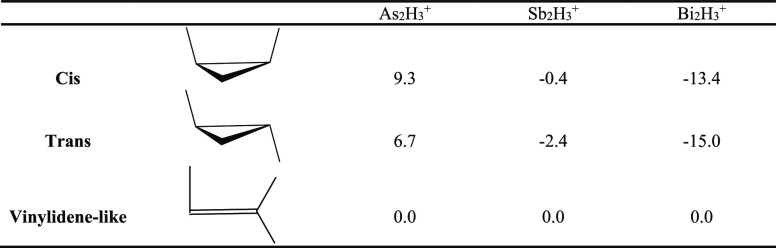
Relative Energies (in kcal/mol) for the E_2_H_3_^+^ Systems (E = As, Sb, Bi)^a^

a Geometries were optimized with the CCSD(T)/cc-pVQZ-PP method.

For Sb_2_H_3_^+^, a staggered minimum was located by the four DFT methods, but it seems to be not viable since there is a very small energy barrier (<0.2 kcal/mol predicted by MN15 and ωB97X-D) to depart to the trans structure. In fact, the high-level CCSD(T) method predicts that no staggered minimum exists, but instead a staggered transition state directly falling to the trans minimum. A planar-cis structure is found for the As_2_H_3_^+^ and Sb_2_H_3_^+^ systems. However, this structure should have little chemical significance because it is a second-order saddle point and has very high energy (>50 kcal/mol above the global minimum).

We have also tried to optimize *C*_2*v*_ structures between two mirror vinylidene structures using the DFT and CCSD(T) methods. However, these *C*_2*v*_ structures are either second-order saddle points with very high energies (76, 76, and 97 kcal/mol above vinylidene-like for E = As, Sb, and Bi, respectively) or lead to dissociation, depending on the electron configurations. Thus, we do not discuss these *C*_2*v*_ structures in the text but report the high-lying geometries in the Supporting Information.

### Proton Affinity

The proton affinity (PA) is an important property for molecules and atoms and is related to the basicity in the gas phase. The study of proton affinities will provide useful information with respect to structure, stability, and bonding. Using the results for E_2_H_3_^+^ (E = As, Sb, Bi) in the present paper and the E_2_H_2_ results in a previous paper,^19^ we can directly obtain the proton affinities for E_2_H_2_. At the CCSD(T)/cc-pVQZ-PP level of theory, the adiabatic proton affinities are predicted to be 180 kcal/mol for As_2_H_2_, 186 kcal/mol for Sb_2_H_2_, and 192 kcal/mol for Bi_2_H_2_ (Table 5). No experimental results are currently available for these species, and our predictions provide new data for them. As a comparison, these values are close to the experimental PA (192 kcal/mol) for the related molecule N_2_H_2_.^37^ The substantial PA values indicate that the added proton strongly stabilizes the neutral E_2_H_2_ species.

**Table 5:**
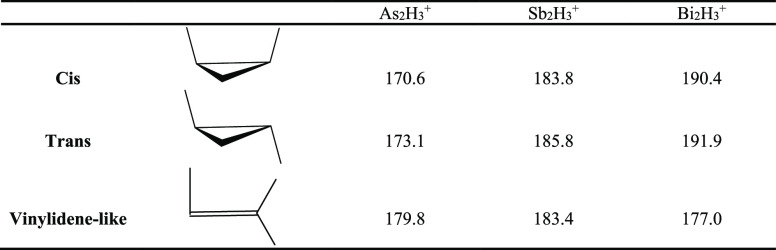
Proton Affinities (in kcal/mol) of E_2_H_2_ Molecules (E = As, Sb, Bi)^a^

a All three neutral E_2_H_2_ molecules have the trans structure lowest in energy. Thus, each proton affinity is computed with respect to the trans E_2_H_2_ structure. All geometries were optimized with the CCSD(T)/cc-pVQZ-PP method.

The NBO analyses may help understand the PA values for E_2_H_2_ (E = As, Sb, Bi). For example, from the NBO analysis, one resonance structure of the trans E_2_H_3_^+^ (E = As, Sb, Bi) species has an occupied E1–H5 bond orbital and an empty lone pair orbital on the E2 (E = As, Sb, Bi) atom, where H5 is the added proton (Figures 1, 3, and 5). The NBO analyses show that the electron distribution via a σ-hyperconjugation effect should occur between the two orbitals. Table 6 reports the second-order perturbation energies *E*^(2)^ of the hyperconjugative σ(As1H5)→LP*(As2) interactions, which are significant, i.e., 194 kcal/mol (As_2_H_3_^+^), 153 kcal/mol (Sb_2_H_3_^+^), and 142 kcal/mol (Bi_2_H_3_^+^). These second-order perturbation values are comparable with the PA values reported above.

**Table 6:** Hyperconjugations and Second-Order Perturbation Energies *E*^(2)^ (in kcal/mol) for Trans HE=EH_2_^+^ Structures (E = As, Sb, Bi) Predicted by NBO Analyses

HAs=AsH_2_^+^		HSb=SbH_2_^+^		HBi=BiH_2_^+^	
hyperconjugation	*E*^(2)^	hyperconjugation	*E*^(2)^	hyperconjugation	*E*^(2)^
σ(As1H5)→LP*(As2)	194.2	σ(Sb1H5)→LP*(Sb2)	152.9	σ(Bi1H5)→LP*(Bi2)	142.0
σ(As1H5)→σ*(As2H4)	2.25	σ(Sb1H5)→σ*(Sb2H4)	3.37	σ(Bi1H5)→σ*(Bi2H4)	2.05
σ(As1As2)→LP*(As2)	6.05	σ(Sb1Sb2)→LP*(Sb2)	4.77	σ(Bi1Bi2)→LP*(Bi2)	4.02

### Observation by Microwave Spectroscopy

The rotational constants and dipole moments for the trans, cis, and vinylidene E_2_H_3_^+^ structures obtained with different computational methods are shown in Table S4. The results for the trans structures from different methods are close to each other. Unlike the E_2_H_2_ molecules, the trans structures for the E_2_H_3_^+^ systems have dipole moments, which are 0.034 D (As_2_H_3_^+^), 0.382 D (Sb_2_H_3_^+^), and 0.461 D (Bi_2_H_3_^+^) at the CCSD(T)/CC-PVQZ-PP level. The small dipole moment for trans As_2_H_3_^+^ indicates that such a structure may be harder to observe in the microwave experiment. All cis E_2_H_3_^+^ structures also have dipole moments, i.e., 1.186 D (As_2_H_3_^+^), 0.640 D (Sb_2_H_3_^+^), and 0.462 D (Bi_2_H_3_^+^). The dipole moment of the vinylidene-like global minimum for As_2_H_3_^+^ is 1.69 D. The large dipole moments of the vinylidene and cis As_2_H_3_^+^ structures suggest that the microwave spectrum may be observable.

## Conclusions

We have used four different DFT methods and the high-level CCSD(T) method to investigate the possible structures of the E_2_H_3_^+^ (E = As, Sb, Bi) compounds. With substantial PAs (>180 kcal/mol), those protonated cations should be viable species. Overall, we report seven stationary points for As_2_H_3_^+^, eight for Sb_2_H_3_^+^, and six for Bi_2_H_3_^+^. For As_2_H_3_^+^, there are three minima (trans, cis, and vinylidene-like structures), among which the vinylidene isomer is the global minimum. For Sb_2_H_3_^+^, among the same three minima, the trans isomer is the lowest-lying structure. For Bi_2_H_3_^+^, the vinylidene isomer is a transition state, collapsing to the trans global minimum. The present theoretical work should be beneficial in future investigations of the E_2_H_3_^+^ cations.

## Supporting Information Available

Cartesian coordinates of optimized structures, harmonic vibrational frequencies, infrared intensities, dipole moments, and rotational constants for the E_2_H_3_^+^ structures (PDF)
