# Supplementary material for: Structures and Energetics of E2H3+ (E = As, Sb, and Bi) Cations
Source: J Phys Chem A. 2024 Jan 16;128(3):563–71. doi: 10.1021/acs.jpca.3c05945 (PMC10823464; doi:10.1021/acs.jpca.3c05945)
Supplement: Supplementary file 1 — jp3c05945_si_001.pdf [file jp3c05945_si_001.pdf]

Structures and Energetics of $E_2H_3^+$ (E = As, Sb, and Bi) Cations

Shu-Hua Xia,[a] Jihuan He,[a] Zhuoqun Liu,[a] Yunhan Liu,[a] Yan Zhang [a]*,
Yaoming Xie,[b] Mitchell, E. Lahm,[b] Gregory H. Robinson,[b] and Henry F. Schaefer III[b]*

[a]*College of Life and Environmental Sciences, Minzu University of China, Beijing 100081, China*
[b]*Department of Chemistry and Center for Computational Quantum Chemistry, University of Georgia, Athens, GA 30602, USA*

Supporting Information

Table of Contents

Table S1. Cartesian Coordinates of All Optimized Structures at MN15/cc-pVTZ-PP level.

Table S2. Cartesian Coordinates of All Optimized Structures at CCSD(T)/cc-pVQZ-PP level.

Table S3. Harmonic vibrational frequencies (cm^{-1}) and their infrared intensities (km/mol, in parentheses) for $E_2H_3^+$ structures.

Table S4. Dipole moments (μ, in Debye) and Rotational Constants (A, B, and C, in GHz) for the trans and cis structures for $E_2H_3^+$ (E = As, Sb, Bi), as well as the vinylidene structure for $As_2H_3^+$.

Table S1. Cartesian Coordinates of All Optimized Structures at MN15/cc-pVTZ-PP level.

$As_2H_3^+$ system

vinylidene-like

As	0.02121700	1.06585300	0.00000000
As	0.02121700	-1.14991600	0.00000000
H	-1.17501000	1.95132800	0.00000000
H	-1.49865100	-1.04511800	0.00000000
H	1.27331500	1.86785400	0.00000000

trans

As	0.00000000	1.17669600	-0.02125400
As	0.00000000	-1.17669600	-0.02125400
H	1.51906500	1.15456000	0.06804400
H	-1.51906500	-1.15456000	0.06804400
H	0.00000000	0.00000000	1.26665400

cis

As	0.04557700	0.01765500	1.18543600
As	0.04557700	0.01765500	-1.18543600
H	-1.46945300	0.04776000	1.27921900
H	-1.46945300	0.04776000	-1.27921900
H	-0.06920700	-1.26077400	0.00000000

TS-T-V

As	1.16672600	0.02443400	-0.04833600
As	-1.18908900	-0.05086700	-0.00299800
H	1.23655000	-1.36168800	0.55855700
H	-1.23984500	1.46833900	-0.10648400
H	0.74129600	0.76562500	1.24195900

TS-C-V

As	-1.19279400	-0.04219200	-0.01103800
As	1.17782800	-0.02508800	-0.04451600
H	-1.37424100	1.46784700	0.07252000
H	1.24295700	1.39393500	0.47699400
H	0.62515300	-0.64152500	1.28377200

TS-C-C

As	0.00000000	1.18240200	-0.05901900
As	0.00000000	-1.18240200	-0.05901900

H	0.00000000	1.73955400	1.37409200
H	0.00000000	-1.73955400	1.37409200
H	0.00000000	0.00000000	1.14703900

planar-Cis

As	0.00000000	1.09201400	0.01175700
As	0.00000000	-1.09201400	0.01175700
H	0.00000000	2.13581000	-1.11403000
H	0.00000000	-2.13581000	-1.11403000
H	0.00000000	0.00000000	1.45210600

$Sb_2H_3^+$ system

trans

Sb	0.00000000	-1.36786100	-0.01486900
Sb	0.00000000	1.36786100	-0.01486900
H	1.69802700	1.28081500	0.08573600
H	-1.69802700	-1.28081500	0.08573600
H	0.00000000	0.00000000	1.34516600

Cis

Sb	-1.37793200	-0.03332800	-0.01233300
Sb	1.37793200	-0.03332800	-0.01233300
H	1.38492400	1.66525500	-0.04031600
H	-1.38492400	1.66525500	-0.04031600
H	0.00001200	0.06898600	1.33862700

vinylidene-like

Sb	0.01597500	-1.32696300	0.00000000
Sb	0.01597500	1.26119400	0.00000000
H	1.36876900	2.23286200	0.00000000
H	-1.67857700	-1.13535900	0.00000000
H	-1.31969300	2.25671200	0.00000000

staggered

Sb	-1.38733000	-0.03348400	0.00131200
Sb	1.36002600	0.00473400	-0.04232100
H	1.34221900	1.16581500	1.19449400
H	-1.38072700	1.66877800	-0.05029100
H	1.43104000	-1.36831700	0.94727900

TS-T-S

Sb	1.36882900	0.00983500	-0.03979100
Sb	-1.38996700	-0.03433900	-0.00124000

H	1.35177200	-1.43938500	0.84441000
H	-1.38542300	1.66772200	-0.06442500
H	1.11167700	1.02136900	1.31261000

TS-C-V

Sb	-1.39048700	-0.03180200	-0.00649300
Sb	1.37889800	-0.01445900	-0.03502500
H	-1.52299200	1.66547100	0.02654100
H	1.29887900	1.51425300	0.70026800
H	0.81510400	-0.82044400	1.39064400

TS-C-C

Sb	0.00000000	1.39324700	-0.04174700
Sb	0.00000000	-1.39324700	-0.04174700
H	0.00000000	2.05737500	1.53488500
H	0.00000000	-2.05737500	1.53488500
H	0.00000000	0.00000000	1.18847100

planar-Cis

Sb	0.00000000	1.27910200	0.00633100
Sb	0.00000000	-1.27910200	0.00633100
H	0.00000000	2.59727800	-1.09817700
H	0.00000000	-2.59727800	-1.09817700
H	0.00000000	0.00000000	1.55064000

$Bi_2H_3^+$ system

trans

Bi	0.00000000	-1.44395200	-0.00922400
Bi	0.00000000	1.44395200	-0.00922400
H	1.77588300	1.33955300	0.07070600
H	-1.77588300	-1.33955300	0.07070600
H	0.00000000	0.00000000	1.38969100

cis

Bi	0.02150600	-0.00793400	-1.45326200
Bi	0.02150600	-0.00793400	1.45326200
H	-1.75263100	-0.03364900	1.43787100
H	-1.75263100	-0.03364900	-1.43787100
H	-0.06474500	1.38426300	0.00000000

vinylidene-like

Bi	0.01054100	-1.39034100	0.00000000
Bi	0.01054100	1.34619400	0.00000000

H	1.37055500	2.43321900	0.00000000
H	-1.75817500	-1.18426800	0.00000000
H	-1.36223000	2.41524800	0.00000000

TS-T-C

Bi	-1.47132200	-0.02112100	-0.00393500
Bi	1.46486800	-0.00355000	-0.02395800
H	-1.56502300	1.75442800	-0.01198600
H	1.16565800	1.40396700	1.03067400
H	0.93504700	-1.11068400	1.29646400

TS-C-C

Bi	0.00000000	1.47448200	-0.02670200
Bi	0.00000000	-1.47448200	-0.02670200
H	0.00000000	2.20486600	1.60017000
H	0.00000000	-2.20486600	1.60017000
H	0.00000000	0.00000000	1.23221400

TS′-C-C

Bi	1.50401000	-0.02390900	0.00000000
Bi	-1.50401000	-0.02390900	0.00000000
H	-1.19158300	1.24548800	1.22542600
H	-1.19158300	1.24548800	-1.22542600
H	1.47616600	1.75008500	0.00006000

Table S2. Cartesian Coordinates of All Optimized Structures at CCSD(T)/cc-pVQZ-PP level.

$As_2H_3^+$ system

vinylidene-like

As	-1.05978935	-0.00367121	0.00000000
As	1.17506446	-0.05246887	0.00000000
H	-1.92756858	1.20927074	0.00000000
H	1.12887797	1.47350401	0.00000000
H	-1.87429850	-1.25068668	0.00000000

trans

As	-0.00268889	1.18034826	-0.02347323
As	0.00268889	-1.18034826	-0.02347323
H	1.52003665	1.17603918	0.05617633
H	-1.52003665	-1.17603918	0.05617633
H	0.00000000	0.00000000	1.26879880

cis

As	-0.01153462	1.18997080	-0.01073840
As	-0.01135055	-1.18650330	-0.02455853
H	-1.52761047	1.29494196	0.07689274
H	-1.52741983	-1.29264270	0.06174229
H	-0.00377853	-0.00576676	1.26871191

TS-T-V

As	1.17119744	0.02782655	-0.04715373
As	-1.19007198	-0.04043293	0.00775910
H	1.23705443	-1.38261070	0.51063044
H	-1.25731724	1.47827825	-0.12706754
H	0.65598935	0.70851583	1.26208772

TS-C-V

As	-1.19397878	-0.03211859	-0.03328056
As	1.18146974	-0.03045214	-0.03999994
H	-1.38227766	1.47638399	0.09748804
H	1.26493940	1.40715632	0.43691579
H	0.56335130	-0.59582058	1.29520267

TS-C-C

As	0.00000000	1.19872335	-0.07887056
As	0.00000000	-1.19872335	-0.07887056
H	0.00000000	1.74061821	1.36298439
H	0.00000000	-1.74061821	1.36298439
H	0.00000000	0.00000000	1.13620434

planar-Cis

As	0.00000000	1.10438116	0.01594882
As	0.00000000	-1.10438116	0.01594882
H	0.00000000	2.14719803	-1.11537544
H	0.00000000	-2.14719803	-1.11537544
H	0.00000000	0.00000000	1.44786524

C_{2v} structure

As	0.00000000	0.00000000	1.04091082
As	0.00000000	0.00000000	-1.05591251
H	0.00000000	1.27451092	1.82462048
H	0.00000000	-1.27451092	1.82462048
H	0.00000000	0.00000000	-2.53401665

$Sb_2H_3^+$ system

trans

Sb	0.00136440	-1.38368902	-0.02057912
Sb	-0.00136440	1.38368902	-0.02057912
H	1.71071263	1.31374913	0.08055538
H	-1.71071263	-1.31374913	0.08055538
H	0.00000000	0.00000000	1.34936449

cis

Sb	-1.39312508	-0.04597953	-0.01712065
Sb	1.39277832	-0.04578626	-0.01756536
H	1.41764896	1.66738665	-0.04086283
H	-1.41833613	1.66720425	-0.03926089
H	0.00008193	0.04428689	1.34371073

vinylidene-like

Sb	0.02193819	-1.37550077	0.00000000
Sb	0.02196774	1.26705649	0.00000000
H	1.40474294	2.21893387	0.00000000
H	-1.68861170	-1.19844384	0.00000000
H	-1.32009517	2.27714625	0.00000000

staggered

Sb	1.38693963	0.01724258	-0.03882371
Sb	-1.40341217	-0.03563472	-0.00242535
H	1.36659005	-1.54442593	0.66276319
H	-1.41787072	1.67950384	-0.07122458
H	0.90908421	0.81774524	1.42806544

TS-T-S

Sb	1.38693963	0.01724258	-0.03882371
Sb	-1.40341217	-0.03563472	-0.00242535
H	1.36659005	-1.54442593	0.66276319
H	-1.41787072	1.67950384	-0.07122458
H	0.90908421	0.81774524	1.42806544

TS-C-V

Sb	-1.40195667	-0.02915603	-0.01237964
Sb	1.39833582	-0.02290851	-0.03470872
H	-1.55372702	1.67908792	0.04770227
H	1.36404791	1.57942286	0.56610721
H	0.76641296	-0.68841424	1.45960888

TS-C-C

Sb	0.00000000	1.43687665	-0.03906810
Sb	0.00000000	-1.43687665	-0.03906810
H	0.00000000	2.08818657	1.55644351
H	0.00000000	-2.08818657	1.55644351
H	0.00000000	0.00000000	1.18157817

Planar-Cis

Sb	0.00000000	1.30445296	0.02124925
Sb	0.00000000	-1.30445296	0.02124925
H	0.00000000	2.60961670	-1.11879880
H	0.00000000	-2.60961670	-1.11879880
H	0.00000000	0.00000000	1.56612210

C_{2v} structure

Sb	0.00000000	0.00000000	0.00000000
Sb	0.00000000	0.00000000	2.47082018
H	0.00000000	1.43338422	-0.88299680
H	0.00000000	-1.43338422	-0.88299680
H	0.00000000	0.00000000	4.12765031

$Bi_2H_3^+$ system

trans

Bi	0.00194665	-1.47235622	-0.01763910
Bi	-0.00194665	1.47235622	-0.01763910
H	1.79469055	1.36412167	0.06666779
H	-1.79469055	-1.36412167	0.06666779
H	0.00000000	0.00000000	1.38850761

cis

Bi	0.03905047	-0.01469606	-1.48224082
Bi	0.03905047	-0.01469606	1.48224082
H	-1.75776762	-0.03954872	1.46242970
H	-1.75776762	-0.03954872	-1.46242970
H	-0.04290470	1.38166356	0.00000000

vinylidene-like

Bi	0.01818213	-1.45710784	0.00000000
Bi	0.02111445	1.35644113	0.00000000
H	1.41918645	2.42209676	0.00000000
H	-1.77269775	-1.24434040	0.00000000
H	-1.36482227	2.43710736	0.00000000

TS-T-C

Bi	-1.51350698	-0.03712505	-0.08748936
Bi	1.47984879	-0.01936692	-0.02057054
H	-1.61716800	1.75494324	0.03672487
H	1.25138850	1.43050421	1.02372374
H	0.92866569	-1.10591547	1.33487029

TS-C-C

Bi	0.00000000	1.57191237	-0.02254343
Bi	0.00000000	-1.57191237	-0.02254343
H	0.00000000	2.25304460	1.64612277
H	0.00000000	-2.25304460	1.64612277
H	0.00000000	0.00000000	1.15641432

C_{2v} structure

Bi	0.00000000	0.00000000	1.30870136
Bi	0.00000000	0.00000000	-1.31463530
H	0.00000000	1.55918323	2.13517205
H	0.00000000	-1.55918323	2.13517205
H	0.00000000	0.00000000	-3.03989597

Table S3. Harmonic vibrational frequencies (cm^{-1}) and their infrared intensities (km/mol, in parentheses) for $E_2H_3^+$ structures.

$As_2H_3^+$ system

**vinylidene-like**	
MN15/cc-pVTZ-PP	210(29),367(2),424(2),637(0),743(9),934(21),2214(17),2334(13),2387(7)
ωB97X-D/cc-pVTZ-PP	246(36),378(2),453(2),649(1),755(6),952(15),2229(14),2315(20),2362(11)
B3LYP/cc-pVTZ-PP	176(34),349(2),427(1),624(0),742(5),935(18),2182(14),2245(18),2303(10)
BP86/cc-pVTZ-PP	169(33),339(1),407(1),601(0),712(5),902(18),2131(12),2169(18),2232(10)
CCSD/cc-pVQZ-PP	382(1),612(1),641(14),790(1),877(4),1001(19),2274(6),2462(22),2508(35)
CCSD(T)/cc-pVQZ-PP	219, 343, 422, 608, 739, 937, 2189, 2320, 2368
**trans**	
MN15/cc-pVTZ-PP	315(0),546(5),719(0),762(69),851(0),1017(182),1506(0),2216(0),2231(45)
ωB97X-D/cc-pVTZ-PP	322(0),557(9),721(0),749(85),855(0),976(173),1527(0),2217(0),2232(33)
B3LYP/cc-pVTZ-PP	298(0),538(9),699((0),722(80),838(0),943(154),1457(0),2172(0),2186(34)
BP86/cc-pVTZ-PP	289(0),506(6),680(0),690(35),809(0),942(170),1429(0),2118(0),2132(29)
CCSD/cc-pVQZ-PP	339(0),774(20),870(70),884(1),946(2),1070(147),1569(2),2281(15),2291(0)
CCSD(T)/cc-pVQZ-PP	304, 540, 708, 736, 841, 985, 1488, 2195, 2208
**cis**	

MN15/cc-pVTZ-PP	306(1),622(6),651(0),738(3),824(7),943(290),1493(1),2232(0),2248(37)
ωB97X-D/cc-pVTZ-PP	315(1),630(7),660(0),738(1),825(5),882(299),1522(1),2229(0),2245(28)
B3LYP/cc-pVTZ-PP	290(0),603(5),643(0),724(2),802(4),857(267),1451(1),2182(0),2197(30)
BP86/cc-pVTZ-PP	281(0),574(4),621(1),694(0),746(4),886(225),1421(1),2131(0),2147(24)
CCSD/cc-pVQZ-PP	332(0),828(10),834(2),868(2),926(8),1002(259),1558(3),2292(0),2306(15)
CCSD(T)/cc-pVQZ-PP	313, 626, 666, 742, 798, 871, 1467, 2252, 2267
**TS-T-V**	
MN15/cc-pVTZ-PP	494i,295(5),507(5),610(10),804(3),935(36),2049(9),2218(12),2242(21)
ωB97X-D/cc-pVTZ-PP	469i,298(5),524(5),616(10),811(2),945(31),2055(13),2219(6),2239(19)
B3LYP/cc-pVTZ-PP	486i,283(3),518(4),606(8),798(1),918(31),1970(14),2173(4),2189(22)
BP86/cc-pVTZ-PP	484i,283(3),492(3),603(8),770(2),874(32),1930(8),2124(5),2139(16)
CCSD(T)/cc-pVQZ-PP	533i, 293, 525, 602, 801, 911, 1986, 2196, 2223
**TS-C-V**	
MN15/cc-pVTZ-PP	544i,290(4),525(16),627(12),716(3),931(36),1964(18),2218(12),2253(19)
ωB97X-D/cc-pVTZ-PP	500i,294(4),542(18),637(11),723(2),938(32),1991(22),2221(6),2247(18)
B3LYP/cc-pVTZ-PP	532i,278(3),526((16),617(9),704(2),909(33),1901(22),2174(6),2196(19)
BP86/cc-pVTZ-PP	545i,277(3),510(11),610(8),674(0),869(36),1854(15),2122(7),2147(12)
CCSD(T)/cc-pVQZ-PP	565i, 286, 531, 620, 705, 908, 1926, 2195, 2229
**TS-C-C**	
MN15/cc-pVTZ-PP	683i,263(1),543(0),590(0),692(6),1375(0),1489(1),2125(0),2126(8)
ωB97X-D/cc-pVTZ-PP	690i,285(1),532(0),604(0),719(3),1300(3),1528(1),2146(1),2148(5)
B3LYP/cc-pVTZ-PP	624i,253(1),523((1),586(0),685(4),1305(1),1466(1),2094(1),2095(5)
BP86/cc-pVTZ-PP	521i,261(0),468(1),586(0),688(5),1166(11),1424(1),2027(2),2028(2)
CCSD(T)/cc-pVQZ-PP	566i,236, 535, 568, 682, 1329, 1451, 2111, 2112
**Planar-Cis**	
MN15/cc-pVTZ-PP	1425i,831i,387(0),576(0),578(1),728(6),1460(42),2063(9),2067(0)
ωB97X-D/cc-pVTZ-PP	1486i,892i,400(0),573(0),584(3),734(4),1505(49),2118(4),2123(0)
B3LYP/cc-pVTZ-PP	1338i,809i,367(0),563(4),571(0),718(3),1420(38),2018(5),2024(0)
BP86/cc-pVTZ-PP	1174i,718i,356(0),540(5),560(0),706(3),1400(35),1932(3),1939(0)
CCSD/cc-pVQZ-PP	1482i,775i,393(0),568(0),627(1),763(5),1509(52),2164(1),2169(0)
CCSD(T)/cc-pVQZ-PP	1286i,678i,363,569,570,724,1447,2037,2040

$Sb_2H_3^+$ system

**trans**	
MN15/cc-pVTZ-PP	208(0),417(4),574(4),671(1),725(55),988(267),1329(14),2012(0),2025(120)
ωB97X-D/cc-pVTZ-PP	217(0),443(4),580(3),677(0),708(87),918(264),1373(15),1980(0),1992(110)
B3LYP/cc-pVTZ-PP	198(0),420(2),553(3),657(0),692(52),929(255),1292(13),1926(0),1938(105)
BP86/cc-pVTZ-PP	193(0),393(2),536(2),632(0),647(25),953(238),1278(7),1873(0),1885(88)
CCSD/cc-pVQZ-PP	219(0),439(4),590(3),680(0),720(84),958(286),1335(11),2088(0),2099(86)
CCSD(T)/cc-pVQZ-PP	200, 413, 560, 656, 699, 951, 1294, 1969, 1980

**cis**	
MN15/cc-pVTZ-PP	203(0),498(3),526(0),589(13),742(13),936(343),1307(16),2033(0),2046(101)
ωB97X-D/cc-pVTZ-PP	213(0),511(4),523(0),597(8),743(12),863(384),1362(16),1979(1),1991(93)
B3LYP/cc-pVTZ-PP	194(0),487(2),516(0),585(8),725(10),890(332),1277(14),1942(0),1954(91)
BP86/cc-pVTZ-PP	189(0),471(2),503(1),561(7),677(7),931(288),1264(8),1894(0),1906(76)
CCSD/cc-pVQZ-PP	214(0),505(4),541(0),604(7),761(12),903(405),1322(13),2100(0),2111(75)
CCSD(T)/cc-pVQZ-PP	194, 477, 510, 576, 731, 909, 1281, 1977, 1988
**vinylidene-like**	
MN15/cc-pVTZ-PP	63i,239(2),306(3),516(1),592(11),765(38),2015(48),2087(0),2127(2)
ωB97X-D/cc-pVTZ-PP	146(20),247(2),359(3),512(0),596(9),790(33),1912(45),2144(1),2176(1)
B3LYP/cc-pVTZ-PP	43(18),226(1),324(3),508(0),570(8),770(37),1921(42),2039(1),2082(1)
BP86/cc-pVTZ-PP	116(17),221(1),318(2),501(0),546(8),747(35),1878(35),1963(1),2010(0)
CCSD/cc-pVQZ-PP	115(20),243(1),327(3),520(0),600(9),1788(41),2082(37),2205(1),2240(0)
CCSD(T)/cc-pVQZ-PP	69, 217, 305, 477, 572, 763, 1956, 2076, 2108
**staggerred**	
MN15/cc-pVTZ-PP	132(19),215(4),314(3),426(10),605(5),803(56),2005(24),2025(1),2035(78)
ωB97X-D/cc-pVTZ-PP	136(20),229(5),327(3),424(10),618(6),822(53),1978(16),1982(44),2042(35)
B3LYP/cc-pVTZ-PP	143(20),261(14),335(0),410(9),593(5),791(57),1939(29),1940(18),1972(26)
BP86/cc-pVTZ-PP	130(25),257(12),370(5),391(1),571(7),752(50),1889(33),1900(2),1941(8)
**TS-T-S**	
MN15/cc-pVTZ-PP	198i,185(3),339(7),445(8),619(4),809(49),1953(32),2020(9),2032(85)
ωB97X-D/cc-pVTZ-PP	243i,188(2),369(9),441(7),625(2),819(42),1872(39),1961(17),1975(77)
B3LYP/cc-pVTZ-PP	327i,182(2),368(8),446(5),619(2),793(42),1829(35),1931(4),1941(86)
BP86/cc-pVTZ-PP	326i,184(2),348(5),445(5),600(2),754(44),1772(21),1884(3),1894(65)
CCSD(T)/cc-pVQZ-PP	335i, 185, 370, 434, 616, 792, 1855, 1973, 1983
**TS-C-V**	
MN15/cc-pVTZ-PP	362i,187(2),365(18),485(9),570(5),812(39),1849(41),2020(12),2032(85)
ωB97X-D/cc-pVTZ-PP	358i,188(2),369(21),490(7),579(8),809(36),1798(45),1952(34),1980(57)
B3LYP/cc-pVTZ-PP	380i,180(2),374(16),471(7),555(5),791(38),1756(39),1931(10),1944(75)
BP86/cc-pVTZ-PP	388i,183(2),352(10),457(7),531(2),754(41),1752(24),1883(11),1895(55)
CCSD(T)/cc-pVQZ-PP	403i, 183, 373, 471, 556, 793, 1792, 1974, 1985
**TS-C-C**	
MN15/cc-pVTZ-PP	595i,159(0),472(0),474(0),547(15),1354(5),1465(46),1965(5),1969(43)
ωB97X-D/cc-pVTZ-PP	677i,167(0),460(0),477(0),541(11),1287(4),1372(31),1942(5),1948(40)
B3LYP/cc-pVTZ-PP	610i,147(0),460(0),465(0),543(14),1256(4),1366(35),1897(2),1902(36)
BP86/cc-pVTZ-PP	529i,157(0),421(0),464(0),538(13),1248(2),1255(9),1841(0),1844(25)
CCSD/cc-pVQZ-PP	589i,151(0),473(0),539(0),590(25),1355(8),1547(130),2001(1),2003(27)
CCSD(T)/cc-pVQZ-PP	523i, 124, 431, 458, 533, 1261, 1435, 1927, 1930
**planar-cis**	
MN15/cc-pVTZ-PP	1226i,761i,238(0),446(1),468(0),610(14),1284(2),1860(73),1872(13)

ωB97X-D/cc-pVTZ-PP	1301i,828i,253(0),457(1),477(0),602(12)1328(2),1860(69),1872(14)
B3LYP/cc-pVTZ-PP	1104i,718i,227(0),442(1),468(0),610(10),1268(0),1772(61),1785(11)
BP86/cc-pVTZ-PP	939i,638i,224(0),428(2),464(0),611(11),1258(1),1719(42),1732(6)
CCSD/cc-pVQZ-PP	1234i,696i,253(0),463(0),508(0),629(17),1333(3),1916(6),1917(23)
CCSD(T)/cc-pVQZ-PP	1072i, 587i, 227, 443, 458, 594, 1251, 1805, 1813

$Bi_2H_3^+$ system

**trans**	
MN15/cc-pVTZ-PP	147(0),360(5),514(7),590(0),694(23),972(284),1264(33),1872(0),1886(189)
ωB97X-D/cc-pVTZ-PP	152(0),401(3),519(6),604(0),697(43),867(275),1233(39),1837(0),1850(201)
B3LYP/cc-pVTZ-PP	139(0),360(4),496(6),579(0),655(20),894(279),1188(30),1840(0),1851(173)
BP86/cc-pVTZ-PP	136(0),335(4),481(5),557(0),605(11),916(258),1165(20),1803(0),1814(141)
CCSD/cc-pVQZ-PP	153(0),384(2),532(5),606(0),704(44),920(335),1234(31),1964(0),1976(164)
CCSD(T)/cc-pVQZ-PP	137, 356, 497, 578, 678, 929, 1201, 1824, 1835
**cis**	
MN15/cc-pVTZ-PP	144(0),449(0),481(1),528(9),692(8),953(311),1244(34),1902(1),1914(158)
ωB97X-D/cc-pVTZ-PP	149(0),472(0),497(0),555(8),706(9),847(332),1238(40),1939(1),1951(170)
B3LYP/cc-pVTZ-PP	136(0),438(0),470(0),529(7),679(7),901(305),1188(31),1860(0),1871(147)
BP86/cc-pVTZ-PP	133(0),421(0),454(0),506(7),635(5),938(279),1173(21),1824(0),1833(120)
CCSD/cc-pVQZ-PP	150(0),457(0),496(0),544(6),720(9),893(391),1222(33),1980(0),1992(142)
CCSD(T)/cc-pVQZ-PP	133, 425, 456, 511, 685, 915, 1189, 1837, 1847
**vinylidene-like**	
MN15/cc-pVTZ-PP	162i,163(1),245(3),481(1),519(9),713(37),1883(71),1938(3),1982(16)
ωB97X-D/cc-pVTZ-PP	192i,170(1),258(3),473(1),505(8),713(35),1837(76),1876(2),1915(18)
B3LYP/cc-pVTZ-PP	229i,154(1),257(3),461(1),488(8),708(37),1852(61),1862(4),1908(15)
BP86/cc-pVTZ-PP	209i,151(0),251(2),451(1),467(7),683(35),1803(1),1821(48),1855(14)
CCSD/cc-pVQZ-PP	162i,165(1),264(3),479(1),537(8),733(45),1965(69),2066(2),2113(10)
CCSD(T)/cc-pVQZ-PP	136i, 144, 248, 424, 497, 704, 1820, 1904, 1944
**TS-T-C**	
MN15/cc-pVTZ-PP	267i,126(2),285(38),449(3),522(2),768(26),1751(90),1854(76),1899(93)
ωB97X-D/cc-pVTZ-PP	241i,125(1),336(29),453(2),529(7),770(25),1712(104),1860(33),1874(145)
B3LYP/cc-pVTZ-PP	218i,122(1),303(23),418(3),498(3),750(30),1726(81),1828(65),1865(83)
BP86/cc-pVTZ-PP	236i,125(1),234(20),423(2),487(1),718(32),1696(59),1773(51),1832(65)
CCSD(T)/cc-pVQZ-PP	235i, 122, 294, 419, 500, 752, 1726, 1813, 1841
**TS-C-C**	
MN15/cc-pVTZ-PP	532i,112(0),433(1),460(0),513(16),1257(7),1401(74),1861(13),1867(71)
ωB97X-D/cc-pVTZ-PP	610i,115(0),440(1),449(0),522(13),1242(8),1452(65),1910(17),1919(83)
B3LYP/cc-pVTZ-PP	571i,96(0),405(1),435(0),496(15),1194(6),1379(69),1827(9),1833(69)
BP86/cc-pVTZ-PP	533i,102(0),385(1),424(0),494(15),1200(3),1310(34),1779(3),1783(49)
CCSD/cc-pVQZ-PP	508i,99(0),480(0),487(0),546(38),1277(6),1500(145),1885(0),1888(48)

CCSD(T)/cc-pVQZ-PP	456i, 66, 366, 401, 492, 1138, 1436, 1808, 1812
**TS'-C-C**	
MN15/cc-pVTZ-PP	301i,118(0),387(20),419(2),495(21),732(11),1797(93),1798(111),1916(72)
ωB97X-D/cc-pVTZ-PP	218i,117(0),430(14),441(3),529(29),757(11),1763(103),1767(130),1966(74)
B3LYP/cc-pVTZ-PP	258i,112(0),396(16),400(2),498(23),724(12),1769(90),1771(101),1881(64)
BP86/cc-pVTZ-PP	297i,116(1),372(21),384(2),482(14),669(16),1713(67),1720(89),1845(53)
CCSD(T)/cc-pVQZ-PP	257i, 115, 386, 403, 481, 731, 1769, 1771, 1850

Table S4. Dipole moments (μ, in Debye) and Rotational Constants (A, B, and C, in GHz) for the trans and cis structures for $E_2H_3^+$, as well as the vinylidene structure for $As_2H_3^+$.

**trans**	Rotational Constants (A, B, and C, in GHZ)	Dipole Moments (|μ|, in Debye)
**MN15/cc-pVTZ-PP**		
Trans (HAs=AsH)-H^+	80.16, 2.41, 2.38	0.144
Trans (HSb=SbH)-H^+	66.02, 1.11, 1.10	0.454
Trans (HBi=BiH)-H^+	60.53, 0.58, 0.58	0.669
**ωB97X-D/cc-pVTZ-PP**		
Trans (HAs=AsH)-H^+	79.89, 2.42, 2.39	0.071
Trans (HSb=SbH)-H^+	65.04, 1.10, 1.10	0.367
Trans (HBi=BiH)-H^+	59.55, 0.58, 0.57	0.577
**B3LYP/cc-pVTZ-PP**		
Trans (HAs=AsH)-H^+	78.68, 2.32, 2.29	0.079
Trans (HSb=SbH)-H^+	64.81, 1.07, 1.06	0.371
Trans (HBi=BiH)-H^+	59.45, 0.55, 0.55	0.575
**BP86/cc-pVTZ-PP**		
Trans (HAs=AsH)-H^+	78.68, 2.32, 2.29	0.019
Trans (HSb=SbH)-H^+	64.06, 1.07, 1.06	0.307
Trans (HBi=BiH)-H^+	58.94, 0.55, 0.55	0.496
**CCSD(T)/cc-pVQZ-PP**		
Trans (HAs=AsH)-H^+	80.82, 2.38, 2.35	0.034
Trans (HSb=SbH)-H^+	65.98, 1.09, 1.08	0.382
Trans (HBi=BiH)-H^+	61.36, 0.56, 0.56	0.461

**cis**	Rotational Constants (A, B, and C, in GHZ)	Dipole Moments (|μ|, in Debye)
**MN15/cc-pVTZ-PP**		
Cis (HAs=AsH)-H^+	81.34, 2.35, 2.31	0.782
Cis (HSb=SbH)-H^+	66.41, 1.09, 1.08	0.461

Cis (HBi=BiH)-H^+	61.11, 0.57, 0.57	0.832
**ωB97X-D/cc-pVTZ-PP**		
Cis (HAs=AsH)-H^+	80.87, 2.38, 2.35	0.981
Cis (HSb=SbH)-H^+	65.37, 1.09, 1.08	0.498
Cis (HBi=BiH)-H^+	60.08, 0.57, 0.56	0.714
**B3LYP/cc-pVTZ-PP**		
Cis (HAs=AsH)-H^+	80.50, 2.31, 2.28	0.875
Cis (HSb=SbH)-H^+	65.21, 1.06, 1.05	0.428
Cis (HBi=BiH)-H^+	59.85, 0.55, 0.55	0.748
**BP86/cc-pVTZ-PP**		
Cis (HAs=AsH)-H^+	79.34, 2.28, 2.25	0.859
Cis (HSb=SbH)-H^+	64.42, 1.05, 1.04	0.384
Cis (HBi=BiH)-H^+	59.28, 0.55, 0.55	0.666
**CCSD(T)/cc-pVQZ-PP**		
Cis (HAs=AsH)-H^+	81.41, 2.35, 2.32	1.186
Cis (HSb=SbH)-H^+	66.22, 1.07, 1.06	0.640
Cis (HBi=BiH)-H^+	61.56, 0.56, 0.56	0.462

**vinylidene-like**	Rotational Constants (A, B, and C, in GHZ)	Dipole Moments (|μ|, in Debye)
**MN15/cc-pVTZ-PP**		
Vinylidene (HAs=AsH)-H^+	94.95, 2.62, 2.55	1.205
**ωB97X-D/cc-pVTZ-PP**		
Vinylidene (HAs=AsH)-H^+	94.53, 2.66, 2.59	1.381
**B3LYP/cc-pVTZ-PP**		
Vinylidene (HAs=AsH)-H^+	94.05, 2.59, 2.52	1.293
**BP86/cc-pVTZ-PP**		
Vinylidene (HAs=AsH)-H^+	92.74, 2.57, 2.50	1.314
**CCSD(T)/cc-pVQZ-PP**		
Vinylidene (HAs=AsH)-H^+	95.15, 2.59, 2.52	1.689
